# Emergency Contraception Knowledge, Attitudes, and Barriers Among Men: A Cross-Sectional Study

**DOI:** 10.7759/cureus.51937

**Published:** 2024-01-09

**Authors:** Hidar Alibrahim, Haidara Bohsas, Sarya Swed, Mohamad Nour Nasif, Abdelmonem Siddiq, Haidara Msallam, Yazan Khair Eldien Jabban, Mohammad Badr Almoshantaf, Hira A Jawed, Moudar Aswad, Nadim Hallak, Razan Kasem, Bisher Sawaf, Ibrahim Elbialy, Ihab Gebaly Mohammed Gabr, Reem Rizk Abazid, Farida Munawar, Azza Bakr Ahmed, Nisrin Moustafa Elsaadouni, Noha Youssef Shalaby, Wael Hafez

**Affiliations:** 1 Department of Internal Medicine, University of Aleppo, Aleppo, SYR; 2 Faculty of Medicine, University of Aleppo, Aleppo, SYR; 3 Department of Medicine, University of Aleppo, Aleppo, SYR; 4 Department of Laboratory Medicine, Faculty of Medicine, University of Aleppo, Aleppo, SYR; 5 Faculty of Pharmacy, Mansoura University, Mansoura, EGY; 6 Faculty of Medicine, Tishreen University, Latakia, SYR; 7 Department of Internal Medicine, Faculty of Medicine, Damascus University, Damascus, SYR; 8 Department of Neurosurgery, Ibn Al-Nafees Hospital, Damascus, SYR; 9 Department of Internal Medicine, Aga Khan Health Services, Karachi, PAK; 10 Department of Internal Medicine, Faculty of Medicine, Syrian Private University, Damascus, SYR; 11 Department of General Medicine, Burjeel Hospital, Abu Dhabi, ARE; 12 Department of Critical Care, Burjeel Medical City, Abu Dhabi, ARE; 13 Department of Obstetrics and Gynecology, New Medical Centre (NMC) Royal Hospital, Abu Dhabi, ARE; 14 Department of Internal Medicine, Ain Shams General Hospital, Ain Shams, EGY; 15 Department of Internal Medicine, NMC Royal Hospital, Khalifa City, ARE; 16 Department of Psychiatry, Faculty of Medicine, Mansoura University, Mansoura, EGY; 17 Department of Physiatry, Mediclinic Hospital, Abu Dhabi, ARE; 18 Department of Internal Medicine, National Research Centre, Cairo, EGY; 19 Department of Internal Medicine, New Medical Centre (NMC) Royal Hospital, Abu Dhabi, ARE

**Keywords:** cross-sectional, family planning., reproductive health, syria, emergency contraception

## Abstract

Background

Emergency contraception (EC) plays a pivotal role in the prevention of unintended pregnancies following unprotected sexual intercourse. Men's awareness regarding emergency contraception is pivotal for informed decision-making and for enhancing reproductive health in this context. This study investigated Syrian men's awareness and perspectives on emergency contraception to inform diverse reproductive health initiatives.

Methods

We conducted a cross-sectional study in Syria, from June 2022 and April 2023. Our study included male participants aged 18 years or older who held Syrian nationality and volunteered to participate. The data collection involved administering a questionnaire comprising three sections (knowledge, attitude, and barrier assessment), encompassing a total of 30 questions. Data analysis was performed using the Statistical Package for Social Sciences (SPSS) (IBM SPSS Statistics, Armonk, NY).

Results

Most participants were aged 18-25 (65.7%) and single (75.4%) and held a university degree (79.3%). The knowledge of emergency contraception was low (36.1%), with the Internet and social media (77.5%) being the primary sources of information. While 89% held positive attitudes toward emergency contraception, only 37.3% supported nonprescription availability. Age, income, and desire for children were associated with knowledge, attitudes, and the use of emergency contraception. Men aged 26-35 exhibited the highest positive attitude (8.11±1.83). Those desiring no children showed higher attitude scores (7.42±2.04). Income was positively associated with knowledge (adjusted odds ratio {AOR}=1.75 and confidence interval {CI}=1.02-2.99) and emergency contraception use (AOR=2.87 and CI=1.27-6.48).

Conclusion

This study underscores the knowledge gap regarding emergency contraception in Syrian men. Despite positive attitudes, awareness remains limited, particularly among those of childbearing age. Targeted education and improved accessibility to emergency contraception can enhance its use among men, particularly in those with low socioeconomic status and younger age groups.

## Introduction

Unintended pregnancies, marked by their occurrence without deliberate planning regarding timing or conception, are a significant global concern. They have substantial implications, contributing to both physical and mental health challenges for both mothers and infants [1]. Furthermore, they often lead to an increase in abortion rates, accompanied by associated medical complications, and are a known risk factor for postpartum depression in developing countries. Notably, in 2017, roughly half of all pregnancies worldwide were classified as unwanted [2,3].

Family planning, defined as the capacity to regulate the number of children and spacing between childbirths [4], plays a pivotal role in curbing unintended pregnancies. Contraceptives encompass a wide array of methods, including condoms that prevent sperm from reaching the ovum and fertilization, hormonal approaches (pills or injections) that alter the ovulatory cycle, intrauterine devices (IUDs), and surgical interventions, such as tubal ligation for women and vasectomy for men. Contraceptive methods vary in terms of administration mode, timing, efficacy, and regional prevalence [5]. For instance, injectable contraceptives are commonly used in Africa, whereas they are less frequently used in developed countries [6].

Emergency contraception (EC) is a critical component of family planning, defined as the use of a device or medication within the first 72 hours after unprotected sexual intercourse to prevent conception [7]. Although no contraceptive method is 100% foolproof, emergency contraception has proven to be significantly effective. It comprises two primary methods: oral contraceptive pills and copper intrauterine devices (IUDs), with IUD implantation feasible within five days of unprotected intercourse. Oral contraceptive pills include various options, such as levonorgestrel, mifepristone, and ulipristal acetate [7].

Despite the importance of contraception, there remains a knowledge gap, particularly evident in Africa, where only 29.4% of women aged 15-49 years use these methods [8]. Research indicates that women with a higher education and economic background are more receptive to emergency contraception. However, educating men about EC is equally vital, as their support can significantly impact positive reproductive healthcare outcomes for families. Equipping men with accurate information empowers them to actively participate in decision-making and facilitates accessibility to EC [9].

In some regions, such as Turkey and Saudi Arabia, there is limited knowledge and awareness of EC among men, as emergency contraceptive usage is often perceived as a female-specific health concern [10,11]. In Muslim-majority countries such as Syria, cultural and religious challenges surrounding termination of pregnancy (TOP) exist due to strong religious condemnation in Islam, the predominant religion in the region. Consequently, EC has emerged as one of the primary options for preventing unwanted pregnancies and TOP [12].

Furthermore, we identified a significant gap in the literature, specifically in the Syrian population, regarding EC awareness. This study aimed to address this knowledge deficiency and offer fresh insights into the role of men in family reproductive decision-making. These findings can inform future strategies to increase awareness among the population and shape healthcare policies aimed at improving reproductive health outcomes.

## Materials and methods

Study design and setting

A cross-sectional study was conducted in Syria between June 2022 and April 2023 to assess the knowledge, attitudes, and barriers related to emergency contraception (EC) among Syrian men. The participants who met the inclusion criteria were males aged 18 years or older with Syrian nationality who willingly participated in the study. The exclusion criteria included individuals under the age of 18 years, females, non-Syrian nationals, those who declined participation, and those who had limited mental or language capacity. All participants were informed of the study's objectives, their right to abstain from participation, the protection of their privacy and data, and the inclusion of only fully completed surveys for analysis. The questionnaire employed in this study was adapted from prior research and included a comprehensive and validated scale [11]. The scale was translated into Arabic to ensure complete comprehension by the participants. The translated questionnaire was subjected to rigorous scrutiny by language experts and culturally adept individuals to ensure that the intended meaning was preserved. To reach a diverse cross section of the Syrian male population, a robust strategy for survey dissemination was devised. A meticulously designed Google Forms (Google, Inc., Mountain View, CA) survey was used to ensure that the participants could easily access and complete the survey. The survey was shared across various social media platforms including Facebook, WhatsApp, and Telegram.

Measures

The evaluation was conducted using a questionnaire divided into three parts consisting of 30 questions/statements.

Sociodemographic Variables

This section comprised seven questions concerning the participants' sociodemographic information, including age, level of education, economic status, work status, marital status, number of children, and recent willingness to have children.

Knowledge Assessment

The participants' knowledge of emergency contraception was evaluated using a series of questions and statements. These questions aimed to determine whether the participants were aware of EC, whether they understood its use within three days of unprotected intercourse, and the depth of their knowledge of EC. If the participants answered affirmatively to being aware of EC, additional questions were asked to assess their knowledge in more detail. These questions covered topics such as when women can use EC, what actions can be taken to prevent pregnancy, the correct timing for its use, its availability, prerequisites for its use, and the sources from which they acquired knowledge about EC.

Attitude Assessment

The participants' attitudes toward emergency contraception were assessed through a series of statements and questions. They were asked to respond with "yes," "no," or "do not know" to these statements, with each statement reflecting a different aspect of attitudes toward EC. A higher score (>5) was considered indicative of a positive or favorable attitude toward EC use, whereas a lower score (≤5) indicated a less favorable or negative attitude.

Barriers Assessment

The study also examined the perceived barriers to the use of emergency contraception. The participants were asked about various factors that might discourage them from using EC, including religious or cultural factors, difficulties in access, concerns about drug side effects, and cost-related issues.

Pilot study

A random sample of 50 men from the general population participated in a pilot study to assess the effectiveness and clarity of the survey. Based on these responses, the questionnaire was revised and improved accordingly. Subsequently, a pilot test was conducted with an additional 50 participants to assess the survey's validity. This pilot study demonstrated high levels of internal consistency, with Cronbach's alpha values ranging from 0.712 to 0.861. Following the successful pilot study, the survey was finalized for publication.

Ethical consideration

Ethical approval for this study was obtained from the University of Aleppo Ethics Committee (approval number: J/55-34). The participants were provided with a link to the survey, and informed consent was obtained before they completed the questionnaire. Detailed research materials were made available to the participants on the following page, and they completed the questionnaire at their own pace. The survey typically took between five and 12 minutes to complete, and all responses were securely stored in an online database.

Statistically analysis

Data analysis was conducted using the Statistical Package for Social Sciences (SPSS) version 23 (IBM SPSS Statistics, Armonk, NY). We calculated descriptive statistics, including frequencies and percentages, sociodemographic data, sources of education on emergency contraceptive pills (ECP), and responses related to ECP knowledge. The chi-squared test was used to examine associations and determine statistical significance. Univariate analyses were performed using descriptive statistics. To identify factors potentially linked to EC awareness and usage, we conducted a multivariable logistic regression. When analyzing factors influencing EC usage, we considered the number of participants with accurate information regarding contraceptive use within three days of unprotected intercourse. Statistical significance was set at P<0.05.

## Results

Sociodemographic characteristics of the study participants

A total of 826 men participated in the study. Most participants (65.7%, n=537) were within the age range of 18-25 years, and a significant portion (75.4%, n=623) were single. Notably, 79.3% (n=655) of the participants held a university degree, while only 0.8% had completed primary school. Furthermore, the majority (n=651, 78.8%) of the participants reported having no children. In terms of income, 49.5% of the participants fell into the moderate-income category, and the largest subgroup (n=376, 45.5%) indicated that they were not currently employed. For a more comprehensive overview of the demographic characteristics, please refer to Table 1.

**Table 1:** Sociodemographic characteristics of the study population The data has been represented as N and %

		Frequency, N	Percentage, %
Age	18-25	537	65.7%
	26-35	149	18.2%
	36-45	66	8.1%
	46-55	46	5.6%
	56-65	19	2.3%
Marital status	Married	190	23.0%
	Single	623	75.4%
	Divorced	13	1.6%
Number of children	0	651	78.8%
	1	34	4.1%
	2	72	8.7%
	3-5	63	7.6%
	>5	6	0.7%
Current desire for children	No	664	80.4%
	Yes	162	19.6%
Level of education	Primary school	7	0.8%
	Middle school	19	2.3%
	Secondary school	76	9.2%
	University	655	79.3%
	Higher than university (master's or PhD)	69	8.4%
Monthly income	Poor	160	19.4%
	Moderate	409	49.5%
	Good	221	26.8%
	Excellent	36	4.4%
Occupational status	Do not work	376	45.5%
	Retired	10	1.2%
	Private employee	331	40.1%
	Governmental employee	109	13.2%

Knowledge of the participants about emergency contraception among men

In assessing the participants' familiarity with contraceptive methods, it is notable that male condoms and oral contraceptives emerged as the most recognized among male respondents, with 97% and 95.2%, respectively (n=801 and n=786), acknowledging the awareness of these methods (refer to Table 2). Conversely, the awareness of emergency contraception (EC) was less prevalent, with only 36.1% (n=298) of men having prior knowledge of it.

**Table 2:** Knowledge of the participants about EC among men The data has been represented as N and % EC: emergency contraception

		Frequency, N	Percentage, %
If a man has had sexual intercourse, is there anything he can do in the first three days after intercourse to prevent pregnancy?	No	70	8.5%
	Do not know	438	53.0%
	Yes	318	38.5%
Have you ever heard of emergency contraception?	No	551	66.7%
	Yes	275	33.3%
What is the source of knowledge about emergency contraceptive pills (ECP)? Newspapers	No	245	89.1%
	Yes	30	10.9%
What is the source of knowledge about ECP? Friends	No	162	58.9%
	Yes	113	41.1%
What is the source of knowledge about ECP? Family members	No	203	73.8%
	Yes	72	26.2%
What is the source of knowledge about ECP? TV or radio	No	204	74.2%
	Yes	71	25.8%
What is the source of knowledge about ECP? Doctor or family planning specialist	No	109	39.6%
	Yes	166	60.4%
What is the source of knowledge about ECP? Internet or social media	No	62	22.5%
	Yes	213	77.5%
What is the correct timing of ECP?	Do not know	37	13.5%
	<72 hours	229	83.3%
	>72 hours	9	3.3%
What can you ask her to do to prevent pregnancy?	Pray	18	6.5%
	Ask the wife to use emergency contraception	143	52.0%
	Ask the wife to have an intrauterine device (IUD) inserted	75	27.3%
	Ask the wife to take extra birth control pill	32	11.6%
	Ask the wife to have an abortion	4	1.5%
	Ask the wife to use herbal remedies	3	1.1%
Why would you use ECP?	To prevent unwanted pregnancy	244	88.7%
	For birth spacing	20	7.3%
	To prevent abortion	11	4.0%
When can women use ECP?	Forget to take a pill	98	35.6%
	Failed withdrawal ejaculation	55	20.0%
	Failure to use barrier methods	64	23.3%
	Condom break	58	21.1%
Emergency contraceptive pills can be obtained from:	External pharmacy	237	86.2%
	Private hospital	14	5.1%
	Governmental hospital	24	8.7%
Do you need to consult a doctor before using ECP?	No	71	25.8%
	Yes	204	74.2%
Is pregnancy test required before ECP pill?	No	164	59.6%
	Yes	111	40.4%
Have you ever used ECP to prevent pregnancy in the past?	No	235	85.5%
	Yes	40	14.5%

Among those who demonstrated awareness of EC, the primary source of information was digital platforms, such as the Internet and social media, as indicated by 77.5% (n=213) of the respondents. Subsequently, healthcare professionals, including physicians and family planning specialists, constituted the next most commonly cited source, as acknowledged by 60.4% (n=166) of the participants. In contrast, only 8.5% of the men reported newspapers as a source of information. It is pertinent to highlight that a significant majority (83.3%, n=229) correctly identified the critical timeframe for EC administration, recognizing its efficacy within a 72-hour window following unprotected sexual intercourse.

Moreover, more than half of the male respondents (52%, n=143) revealed that they advocated their partners to consider EC as a means of pregnancy prevention. A noteworthy percentage of the participants (74.2%, n=204) emphasized the importance of consulting a medical professional prior to EC use, while a substantial majority (59.6%, n=164) correctly asserted that a pre-administration pregnancy test was unnecessary. Intriguingly, most participants (85.5%, n=235) had not previously utilized EC (Table 3).

**Table 3:** Men's knowledge about different methods of contraception The data has been represented as N and %

			Frequency, N	Percentage, %
Methods of contraception	Male condom	No	25	3.0%
		Yes	801	97.0%
	Implant intrauterine device	No	89	10.8%
		Yes	737	89.2%
	Oral contraceptives	No	40	4.8%
		Yes	786	95.2%
	Injectable hormonal contraception	No	468	56.7%
		Yes	358	43.3%
	Withdrawal methods	No	171	20.7%
		Yes	655	79.3%
	Emergency contraceptive pill	No	528	63.9%
		Yes	298	36.1%

Participants' attitudes and beliefs regarding emergency contraception

Inquiries concerning the decision-making process for emergency contraception (EC) use revealed that the majority of the participants (88.4%, n=730) advocated for shared decision-making involving both partners. However, it is noteworthy that only 37.3% expressed agreement with the concept of making EC available without a prescription, whereas 87.8% (n=725) believed that men should have the ability to purchase EC.

Exploring men's behaviors regarding EC, 68.9% (n=569) indicated their willingness to procure EC for emergency situations in order to keep it readily available at home. Additionally, a substantial majority (82.3%, n=680) expressed their readiness to recommend EC to men facing the risk of an unplanned pregnancy.

When exploring their beliefs concerning EC, it emerged that more than one-third of the men (33.5%, n=277) reported feeling embarrassed about purchasing EC. Furthermore, the side effects of EC were identified as the most frequently cited factor (66.6%, n=550) influencing the decision to use it (see Table 4).

**Table 4:** Participants' attitudes and beliefs regarding emergency contraception (EC) The data has been represented as N and % ECP: emergency contraceptive pills

		Frequency, N	Percentage, %
Affective (feelings)			
The decision to use ECP is ultimately the decision of:	Female partner	54	6.5%
	Male partner	42	5.1%
	Both	730	88.4%
Should ECP be more widely advertised?	No	156	18.9%
	Yes	670	81.1%
Should ECP be available without prescription?	No	518	62.7%
	Yes	308	37.3%
Would you prefer your partner to get the ECP from the pharmacy or clinic?	No	180	21.8%
	Yes	646	78.2%
Men should be able to buy ECP	No	101	12.2%
	Yes	725	87.8%
Behavioral (behavior)			
ECP reduces the chance of pregnancy by up to 75%; would you ask your wife to use it to prevent pregnancy?	No	338	40.9%
	Yes	488	59.1%
I would buy EC to have at home or on hand, just in case of emergencies	No	257	31.1%
	Yes	569	68.9%
Men being able to buy ECP would help to prevent unplanned pregnancies	No	122	14.8%
	Yes	704	85.2%
I would recommend ECP to a man at risk of being involved in an unplanned pregnancy	No	146	17.7%
	Yes	680	82.3%
Cognition (beliefs)			
Do you feel embarrassed to buy?	No	549	66.5%
	Yes	277	33.5%
Religion	No	418	50.6%
	Yes	408	49.4%
Culture	No	420	50.8%
	Yes	406	49.2%
Drug side effects (nausea, vomiting, etc.)	No	276	33.4%
	Yes	550	66.6%
Difficulty to access	No	416	50.4%
	Yes	410	49.6%
Cost	No	403	48.8%
	Yes	423	51.2%

The differences between attitudes scores regarding emergency contraception based on sociodemographic characteristics of the study population

**Figure 1:**
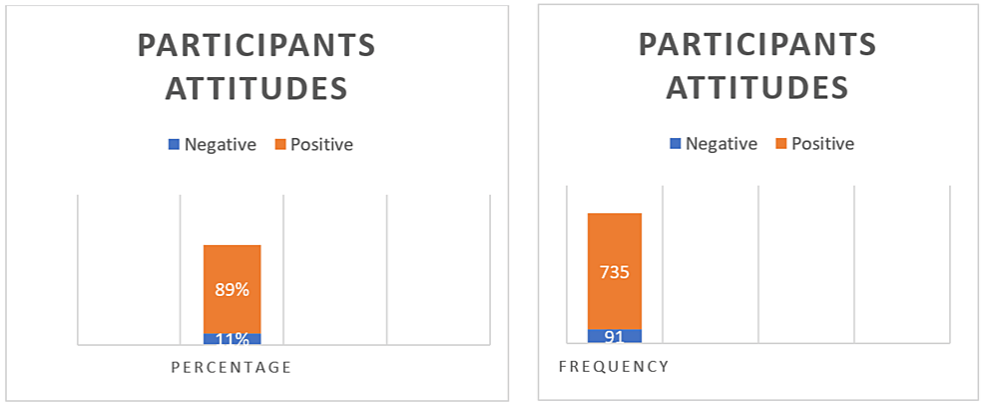
Participants' attitudes and beliefs regarding emergency contraception

In terms of attitudes toward emergency contraception (EC), a substantial 89% of men exhibited a satisfactory outcome, as indicated by a mean attitude score of 7.35±2.04 (see Figure 1 and Table 5). Notably, eight variables demonstrated statistically significant association with attitudes toward EC (P<0.05).

**Table 5:** The mean of attitude score regarding emergency contraception based on sociodemographic characteristics of the study population The data has been represented as mean and standard deviation (SD) (minimum and maximum)

	Mean	Standard deviation	Minimum	Maximum
Attitude	7.35	2.04	0.00	10.00

The participants within the age group of 26-35 exhibited the highest mean attitude score, standing at 8.11±1.83. Additionally, men who did not express a desire for children exhibited a higher attitude score compared to those desiring children, with scores of 7.42±2.04 and 7.06±2.04, respectively. Furthermore, individuals with educational qualifications beyond a university degree displayed the highest attitude scores when compared to their counterparts with varying educational backgrounds, recording a mean score of 8.04±1.86.

Men who did not identify religion as a barrier to EC reported a higher mean attitude score (7.61±1.87). Moreover, those who reported experiencing difficulty accessing EC also presented a higher mean attitude score (7.76±1.82) in contrast to men who did not encounter such access barriers (refer to Table 6).

**Table 6:** The differences between attitude scores regarding emergency contraception based on sociodemographic characteristics of the study population The data has been represented as mean and SD. P-value is significant when <0.05

		Attitude		
		Mean	Standard deviation (SD)	P-value (significant when <0.05)
Age	18-25	7.17	2.01	0.000
	26-35	8.11	1.83	
	36-45	7.12	2.05	
	46-55	7.22	2.55	
	56-65	8.05	2.07	
Marital status	Married	7.42	2.11	0.462
	Single	7.34	2.02	
	Divorced	6.77	2.49	
Number of children	0	7.34	2.01	0.443
	1	7.74	1.97	
	2	7.36	2.18	
	3-5	7.24	2.28	
	<5	6.50	1.76	
Current desire for children	No	7.42	2.04	0.033
	Yes	7.06	2.04	
Level of education	Primary school	5.86	2.04	0.000
	Middle school	8.00	1.45	
	Secondary school	6.49	2.50	
	University	7.37	1.98	
	More than university	8.04	1.86	
Monthly income	Bad	7.46	2.00	0.088
	Moderate	7.20	2.09	
	Good	7.45	2.00	
	Excellent	7.94	1.84	
Occupational status	Do not work	7.24	1.98	0.091
	Retired	8.40	.70	
	Private employee	7.38	2.12	
	Governmental employee	7.52	2.10	
Newspapers	No	7.71	1.90	0.599
	Yes	7.47	2.06	
Friends	No	7.60	1.86	0.279
	Yes	7.79	2.01	
Family members	No	7.68	1.87	0.758
	Yes	7.68	2.07	
TV or radio	No	7.74	1.90	0.438
	Yes	7.52	1.98	
Doctor or family planning specialist	No	7.68	1.89	0.868
	Yes	7.68	1.95	
Internet or social media	No	7.32	2.03	0.108
	Yes	7.78	1.88	
Religion	No	7.61	1.87	0.000
	Yes	7.07	2.18	
Culture	No	7.58	1.96	0.000
	Yes	7.10	2.10	
Drug side effects (nausea, vomiting, etc.)	No	7.48	2.19	0.032
	Yes	7.28	1.96	
Difficulty to access	No	6.94	2.17	0.000
	Yes	7.76	1.82	
Cost	No	7.15	2.16	0.008
	Yes	7.54	1.91	

Binary logistic regression between baseline characteristics of the study population and knowledge of the respondents relating to emergency contraception

Of the 13 variables examined, only age and monthly income demonstrated statistically significant associations with the prediction of knowledge regarding emergency contraception (EC) (P<0.05).

Specifically, men aged between 46 and 55 years were found to be 8.7 times less likely than their counterparts aged between 18 and 25 years to possess knowledge about EC (adjusted odds ratio {AOR}=0.16 and confidence interval {CI}=0.36-0.74). Conversely, the participants with a higher monthly income exhibited a greater likelihood of having knowledge about EC than those with lower income levels (AOR=1.75 and CI=1.02-2.99) (Table 7).

**Table 7:** Binary logistic regression between baseline characteristics of the study population and knowledge of the respondents relating to emergency contraception The data has been represented as N, %, COR, and AOR. P-value is significant when <0.05 COR, crude odds ratio; AOR, adjusted odds ratio

		Knowledge											
		No		Yes									
		Frequency, N	Percentage, %	Frequency, N	Percentage, %	P-value (significant when <0.05)	COR	Lower	Upper	P-value (significant when <0.05)	AOR	Lower	Upper
Age	18-25	379	46.4%	158	19.3%	0.003	-	-	-	0.173	-	-	-
	26-35	110	13.5%	39	4.8%	0.438	0.850	0.565	1.281	0.141	0.640	0.353	1.159
	36-45	58	7.1%	8	1.0%	0.004	0.331	0.154	0.709	0.116	0.409	0.134	1.248
	46-55	43	5.3%	3	0.4%	0.003	0.167	0.051	0.547	0.019	0.164	0.036	0.745
	56-65	19	2.3%	0	0.0%	0.998	0.000	0.000	-	0.998	0.000	0.000	-
Marital status	Married	166	20.1%	24	2.9%	0.000	-	-	-	0.310	-	-	-
	Single	440	53.3%	183	22.2%	0.000	2.877	1.813	4.563	0.159	2.401	0.711	8.111
	Divorced	10	1.2%	3	0.4%	0.293	2.075	0.533	8.079	0.299	2.438	0.454	13.093
Number of children	0	464	56.2%	187	22.6%	0.004	-	-	-	0.113	-	-	-
	1	26	3.1%	8	1.0%	0.514	0.763	0.339	1.717	0.240	2.332	0.569	9.563
	2	59	7.1%	13	1.6%	0.058	0.547	0.293	1.020	0.112	3.182	0.764	13.258
	3-5	61	7.4%	2	0.2%	0.001	0.081	0.020	0.336	0.372	0.410	0.058	2.903
	>5	6	0.7%	0	0.0%	0.999	0.000	0.000	-	0.999	0.000	0.000	-
Current desire for children	No	491	59.4%	173	20.9%		-	-	-	-	-	-	-
	Yes	125	15.1%	37	4.5%	0.400	0.840	0.560	1.260	0.908	1.029	0.636	1.664
Level of education	Primary school	7	0.8%	0	0.0%	0.000	-	-	-	0.000	-	-	-
	Middle school	19	2.3%	0	0.0%	1.000	1.000	0.000	-	1.000	1.000	0.000	-
	Secondary school	71	8.6%	5	0.6%	0.999	1.000	0.000	-	0.999	1.000	0.000	-
	University	484	58.6%	171	20.7%	0.999	1.000	0.000	-	0.999	1.000	0.000	-
	More than university	35	4.2%	34	4.1%	0.999	1.000	0.000	-	0.999	1.000	0.000	-
Monthly income	Bad	129	15.6%	31	3.8%	0.025	-	-	-	0.172	-	-	-
	Moderate	312	37.8%	97	11.7%	0.266	1.294	0.822	2.036	0.321	1.283	0.784	2.098
	Good	151	18.3%	70	8.5%	0.008	1.929	1.189	3.129	0.039	1.754	1.028	2.994
	Excellent	24	2.9%	12	1.5%	0.071	2.081	0.938	4.613	0.212	1.743	0.729	4.170
Occupational status	Do not work	271	32.8%	105	12.7%	0.626	-	-	-	0.238	-	-	-
	Retired	10	1.2%	0	0.0%	0.999	0.000	0.000	-	0.999	0.000	0.000	-
	Private employee	252	30.5%	79	9.6%	0.220	0.809	0.577	1.135	0.811	0.951	0.631	1.433
	Governmental employee	83	10.0%	26	3.1%	0.400	0.808	0.493	1.326	0.080	1.893	0.927	3.867
Religion	No	320	38.7%	98	11.9%	-	-	-	-	-	-	-	-
	Yes	296	35.8%	112	13.6%	0.187	1.236	0.903	1.691	0.421	1.164	0.804	1.686
Culture	No	316	38.3%	104	12.6%	-	-	-	-	-	-	-	-
	Yes	300	36.3%	106	12.8%	0.657	1.074	0.785	1.469	0.573	1.114	0.766	1.619
Drug side effects (nausea, vomiting, etc.)	No	191	23.1%	85	10.3%	-	-	-	-	-	-	-	-
	Yes	425	51.5%	125	15.1%	0.012	0.661	0.478	0.914	0.090	0.735	0.515	1.049
Difficulty to access	No	309	37.4%	107	13.0%	-	-	-	-	-	-	-	-
	Yes	307	37.2%	103	12.5%	0.843	0.969	0.708	1.325	0.393	1.189	0.799	1.768
Cost	No	291	35.2%	112	13.6%	-	-	-	-	-	-	-	-
	Yes	325	39.3%	98	11.9%	0.128	0.783	0.572	1.072	0.507	0.872	0.583	1.305
Attitude grade	Negative	75	9.1%	16	1.9%	-	-	-	-	-	-	-	-
	Positive	541	65.5%	194	23.5%	0.071	1.681	0.956	2.955	0.216	1.464	0.801	2.678
Constant		-	-	-	-	-	-	-	-	0.999	0.000	-	-

Binary logistic regression between baseline characteristics of the study population and attitude of the respondents relating to emergency contraception

Four predictors, namely, age, current desire to have children, religion as a barrier, and difficulty in access, demonstrated statistically significant correlations with attitudes toward emergency contraception (EC) (P<0.05).

The participants aged between 26 and 35 years exhibited a noteworthy 5.37-fold increase in the likelihood of holding a positive attitude toward EC compared to those in the 18-25 age bracket (adjusted odds ratio {AOR}=5.37 and confidence interval {CI}=1.76-16.37). Conversely, individuals expressing a desire to have children displayed reduced odds of maintaining a positive attitude toward EC compared to those who did not (AOR=0.47 and CI=0.26-0.84).

Furthermore, the participants who identified religion as a barrier to EC were 0.52 times less likely to maintain a positive attitude (AOR=0.52 and CI=0.3-0.87). Conversely, the respondents reporting difficulty in accessing EC demonstrated higher odds of maintaining a positive attitude (AOR=2.76 and CI=1.54-4.96) (see Table 8).

**Table 8:** Binary logistic regression between baseline characteristics of the study population and attitude of the respondents relating to emergency contraception The data has been represented as N, %, COR, and AOR. P-value is significant when <0.05 COR, crude odds ratio; AOR, adjusted odds ratio

		Attitude grade											
		Negative		Positive									
		Frequency, N	Percentage, %	Frequency, N	Percentage, %	P-value (significant when <0.05)	COR	Lower	Upper	P-value (significant when <0.05)	AOR	Lower	Upper
Age	18-25	64	7.8%	473	57.9%	0.122	-	-	-	0.063	-	-	-
	26-35	7	0.9%	142	17.4%	0.014	2.745	1.230	6.124	0.003	5.371	1.761	16.374
	36-45	9	1.1%	57	7.0%	0.687	0.857	0.405	1.814	0.165	2.553	0.680	9.591
	46-55	7	0.9%	39	4.8%	0.513	0.754	0.324	1.756	0.182	2.843	0.612	13.196
	56-65	2	0.2%	17	2.1%	0.854	1.150	0.260	5.094	0.576	1.840	0.217	15.617
Marital status	Married	24	2.9%	166	20.1%	0.616	-	-	-	0.465	-	-	-
	Single	65	7.9%	558	67.6%	0.396	1.241	0.753	2.045	0.595	1.496	0.339	6.598
	Divorced	2	0.2%	11	1.3%	0.774	0.795	0.166	3.808	0.326	0.410	0.069	2.424
Number of children	0	68	8.2%	583	70.6%	0.763	-	-	-	0.774	-	-	-
	1	3	0.4%	31	3.8%	0.763	1.205	0.359	4.047	0.902	1.118	0.188	6.641
	2	10	1.2%	62	7.5%	0.373	0.723	0.354	1.476	0.525	0.596	0.121	2.941
	3-5	9	1.1%	54	6.5%	0.350	0.700	0.331	1.480	0.295	0.401	0.072	2.220
	<5	1	0.1%	5	0.6%	0.625	0.583	0.067	5.065	0.999	150196195.567	0.000	-
Current desire for children	No	66	8.0%	598	72.4%	-	-	-	-	-	-	-	-
	Yes	25	3.0%	137	16.6%	0.047	0.605	0.368	0.994	0.012	0.470	0.261	0.849
Level of education	Primary school	1	0.1%	6	0.7%	0.026	-	-	-	0.036	-	-	-
	Middle school	0	0.0%	19	2.3%	0.998	269245807.142	0.000	-	0.998	165911098.015	0.000	-
	Secondary school	17	2.1%	59	7.1%	0.623	0.578	0.065	5.141	0.283	0.254	0.021	3.101
	University	69	8.4%	586	70.9%	0.749	1.415	0.168	11.931	0.861	0.805	0.070	9.242
	More than university	4	0.5%	65	7.9%	0.405	2.708	0.259	28.270	0.942	0.906	0.064	12.748
Monthly income	Bad	17	2.1%	143	17.3%	0.374	-	-	-	0.231	-	-	-
	Moderate	52	6.3%	357	43.2%	0.493	0.816	0.457	1.459	0.937	1.026	0.542	1.944
	Good	20	2.4%	201	24.3%	0.609	1.195	0.605	2.361	0.285	1.500	0.714	3.151
	Excellent	2	0.2%	34	4.1%	0.362	2.021	0.446	9.168	0.101	5.770	0.711	46.855
Occupational status	Do not work	46	5.6%	330	40.0%	0.743	-	-	-	0.836	-	-	-
	Retired	0	0.0%	10	1.2%	0.999	0.000	0.000	-	0.999	301212511.781	0.000	-
	Private employee	32	3.9%	299	36.2%	0.278	1.302	0.808	2.100	0.403	1.293	0.708	2.360
	Governmental employee	13	1.6%	96	11.6%	0.931	1.029	0.534	1.984	0.947	1.034	0.390	2.736
Religion	No	32	3.9%	386	46.7%	-	-	-	-	-	-	-	-
	Yes	59	7.1%	349	42.3%	0.002	0.490	0.311	0.772	0.014	0.520	0.308	0.877
Culture	No	38	4.6%	382	46.2%	-	-	-	-	-	-	-	-
	Yes	53	6.4%	353	42.7%	0.067	0.663	0.426	1.030	0.389	0.797	0.475	1.336
Drug side effects (nausea, vomiting, etc.)	No	31	3.8%	245	29.7%	-	-	-	-	-	-	-	-
	Yes	60	7.3%	490	59.3%	0.889	1.033	0.652	1.637	0.854	0.954	0.580	1.571
Difficulty to access	No	63	7.6%	353	42.7%	-	-	-	-	-	-	-	-
	Yes	28	3.4%	382	46.2%	0.000	2.435	1.525	3.888	0.001	2.767	1.542	4.965
Cost	No	52	6.3%	351	42.5%	-	-	-	-	-	-	-	-
	Yes	39	4.7%	384	46.5%	0.092	1.459	0.940	2.264	0.619	1.155	0.655	2.037
Constant		-	-	-	-	-	-	-	-	0.205	6.128	-	-

Binary logistic regression between baseline characteristics of the study population and using the emergency contraceptive pill

Out of the 13 variables under consideration, only two demonstrated statistically significant correlations with the prediction of emergency contraceptive pill (ECP) utilization (P<0.05).

Men aged-46-55 years were found to be 0.26 times less likely to use ECP compared to their counterparts in the 18-25 age group (adjusted odds ratio {AOR}=0.26 and confidence interval {CI}=0.08-0.83). Conversely, the participants with an excellent monthly income exhibited a notable 2.87-fold increase in the likelihood of using ECP compared to those with lower income levels (AOR=2.87 and CI=1.27-6.48) (Table 9).

**Table 9:** Binary logistic regression between baseline characteristics of the study population and using the emergency contraceptive pill The data has been represented as N, %, COR, and AOR. P-value is significant when <0.05 COR, crude odds ratio; AOR, adjusted odds ratio

		Emergency contraceptive pill											
		No		Yes									
		Frequency, N	Percentage, %	Frequency, N	Percentage, %	P-value (significant when <0.05)	COR	Lower	Upper	P-value (significant when <0.05)	AOR	Lower	Upper
Age	18-25	321	39.3%	216	26.4%	0.000	-	-	-	0.097	-	-	-
	26-35	95	11.6%	54	6.6%	0.379	0.845	0.580	1.230	0.088	0.626	0.366	1.073
	36-45	50	6.1%	16	2.0%	0.013	0.476	0.264	0.857	0.285	0.617	0.255	1.495
	46-55	39	4.8%	7	0.9%	0.002	0.267	0.117	0.607	0.024	0.260	0.081	0.834
	56-65	18	2.2%	1	0.1%	0.016	0.083	0.011	0.623	0.073	0.099	0.008	1.235
Marital status	Married	148	17.9%	42	5.1%	0.000	-	-	-	0.231	-	-	-
	Single	375	45.4%	248	30.0%	0.000	2.330	1.596	3.402	0.538	1.340	0.527	3.406
	Divorced	5	0.6%	8	1.0%	0.004	5.638	1.752	18.143	0.092	3.476	0.816	14.815
Number of children	0	393	47.6%	258	31.2%	0.000	-	-	-	0.245	-	-	-
	1	22	2.7%	12	1.5%	0.614	0.831	0.404	1.708	0.846	1.119	0.360	3.472
	2	53	6.4%	19	2.3%	0.030	0.546	0.316	0.944	0.668	1.275	0.420	3.866
	3-5	57	6.9%	6	0.7%	0.000	0.160	0.068	0.377	0.152	0.376	0.098	1.436
	<5	3	0.4%	3	0.4%	0.608	1.523	0.305	7.605	0.485	2.491	0.192	32.352
Current desire for children	No	429	51.9%	235	28.5%	-	-	-	-	-	-	-	-
	Yes	99	12.0%	63	7.6%	0.406	1.162	0.816	1.655	0.239	1.292	0.843	1.980
Level of education	Primary school	4	0.5%	3	0.4%	0.000	-	-	-	0.000	-	-	-
	Middle school	18	2.2%	1	0.1%	0.042	0.074	0.006	0.911	0.127	0.120	0.008	1.826
	Secondary school	60	7.3%	16	1.9%	0.204	0.356	0.072	1.753	0.470	0.499	0.076	3.288
	University	421	51.0%	234	28.3%	0.696	0.741	0.164	3.340	0.688	0.690	0.113	4.219
	More than university	25	3.0%	44	5.3%	0.289	2.347	0.486	11.340	0.247	3.022	0.465	19.633
Monthly income	Bad	112	13.6%	48	5.8%	0.016	-	-	-	0.055	-	-	-
	Moderate	268	32.4%	141	17.1%	0.309	1.228	0.827	1.822	0.187	1.339	0.868	2.064
	Good	132	16.0%	89	10.8%	0.040	1.573	1.021	2.423	0.060	1.583	0.982	2.555
	Excellent	16	1.9%	20	2.4%	0.005	2.917	1.393	6.109	0.011	2.877	1.277	6.480
Occupational status	Do not work	223	27.0%	153	18.5%	0.046	-	-	-	0.257	-	-	-
	Retired	9	1.1%	1	0.1%	0.086	0.162	0.020	1.291	0.539	0.386	0.019	8.047
	Private employee	222	26.9%	109	13.2%	0.033	0.716	0.526	0.974	0.267	0.808	0.554	1.178
	Governmental employee	74	9.0%	35	4.2%	0.106	0.689	0.439	1.083	0.369	1.338	0.709	2.526
Religion	No	272	32.9%	146	17.7%	-	-	-	-	-	-	-	-
	Yes	256	31.0%	152	18.4%	0.486	1.106	0.833	1.470	0.871	0.973	0.695	1.361
Culture	No	275	33.3%	145	17.6%	-	-	-	-	-	-	-	-
	Yes	253	30.6%	153	18.5%	0.344	1.147	0.863	1.524	0.162	1.273	0.908	1.785
Drug side effects (nausea, vomiting, etc.)	No	166	20.1%	110	13.3%	-	-	-	-	-	-	-	-
	Yes	362	43.8%	188	22.8%	0.110	0.784	0.581	1.056	0.391	0.866	0.623	1.203
Difficulty to access	No	265	32.1%	151	18.3%	-	-	-	-	-	-	-	-
	Yes	263	31.8%	147	17.8%	0.894	0.981	0.738	1.303	0.698	1.074	0.749	1.540
Cost	No	248	30.0%	155	18.8%	-	-	-	-	-	-	-	-
	Yes	280	33.9%	143	17.3%	0.164	0.817	0.615	1.086	0.403	0.855	0.593	1.234
Attitude grade	Negative	68	8.2%	23	2.8%	-	-	-	-	-	-	-	-
	Positive	460	55.7%	275	33.3%	0.024	1.767	1.077	2.902	0.052	1.697	0.995	2.893
Constant		-	-	-	-	-	-	-	-	0.328	0.341	-	-

## Discussion

Emergency contraception (EC) is a crucial intervention for preventing unintended pregnancies following unprotected sexual intercourse, contraceptive failure, or sexual assault [13,14]. Although several studies have focused on women's knowledge of and attitudes toward EC, it is important to acknowledge that male sexual partners also play a significant role in decisions related to EC use [15-19]. This study aimed to explore the knowledge, attitudes, and barriers to EC among Syrian men. The results indicate a significant knowledge gap among Syrian men regarding emergency contraceptives, with only 36% of the participants reporting awareness of EC [20]. This finding is consistent with those of prior studies that have highlighted lower levels of knowledge among men than among women. One contributing factor may be that contraceptive marketing and counseling predominantly target women, leading to a lack of motivation among men to seek information regarding these methods [21,22].

The study also revealed that male condoms and oral contraceptives were the most recognized contraceptive methods among the participants. This aligns with previous research demonstrating that men are more familiar with and prefer condoms and oral contraceptives than other contraceptive options [22-25]. Among those who had heard of EC, the Internet and social media emerged as the primary sources of information, followed by healthcare providers [26,27]. This underscores the essential role of healthcare professionals in disseminating accurate information about EC. However, it is worth noting that the study identified room for improvement in healthcare workers' knowledge of EC, highlighting the need for training in this area [28,29].

A positive aspect of the findings was that a substantial proportion of the participants (around 80%) correctly understood that EC should be administered within 72 hours of unprotected intercourse. In addition, the majority (85.5%) believed that consulting a healthcare provider before using the EC was necessary. These results emphasize the crucial role of healthcare providers in promoting EC use and education [30-32]. Despite limited knowledge, a significant portion of the participants exhibited a positive attitude toward EC. Attitudes were more favorable among middle-aged participants (26-35 years old) and those with university degrees. Education was positively correlated with knowledge levels and, subsequently, attitudes toward EC [33,34]. Binary logistic regression analysis revealed that good monthly income, the lack of religious barriers, and desire to have children were factors associated with more positive attitudes toward EC. The participants who identified religion as a barrier tended to have less favorable attitudes. However, those who reported difficulty accessing EC had more positive attitudes, possibly reflecting greater awareness of its importance in such circumstances [35].

Among the barriers reported, concerns about side effects emerged as the most influential factor affecting EC usage, reported by 66% of the participants [36]. This is consistent with previous research that highlights women's concerns about side effects as a significant factor influencing their decisions regarding EC [37,38]. To enhance reproductive health outcomes, Syrian healthcare providers should receive comprehensive training on emergency contraception (EC) to bridge knowledge gaps and offer patient-centered counseling. Encouraging joint decision-making between couples in family planning is crucial, emphasizing open communication and shared responsibility. Culturally competent care that respects religious beliefs should be prioritized. Proactive discussions about the side effects of EC can alleviate concerns, and tailored educational programs should dispel misconceptions and provide culturally sensitive information. Policymakers should consider making EC more accessible without prescription and launch public health campaigns to increase awareness among Syrian men and women.

Recommendations

The study's findings suggest several recommendations for improving knowledge, attitudes, and barriers related to EC.

Community-Specific Interventions

Conduct further studies involving diverse populations from various cultural and religious backgrounds to identify barriers specific to each community. Tailor educational programs to address these barriers and misconceptions [39].

Healthcare Provider Training

Enhance the education of healthcare providers about different types of emergency contraceptives and their administration. Well-informed providers can offer patient-centered care and play a critical role in promoting EC usage.

Tailored Educational Programs

Develop educational programs tailored to the needs of populations from different cultural and religious backgrounds. These programs should aim to dispel misconceptions, address barriers, and provide accurate information regarding EC.

Strengths and limitations

This study is the first of its kind among Syrian men and provides valuable insights into their knowledge, attitudes, and barriers regarding EC. However, this study has limitations, including a relatively small sample size, nonrandom sampling, and its cross-sectional nature, which does not allow for the establishment of causal relationships.

## Conclusions

This study sheds light on knowledge gaps, attitudes, and barriers related to emergency contraception among Syrian men. We find an overall significant knowledge gap in Syrian men's knowledge. Male condoms and oral contraceptives were the most recognized contraceptive methods among participants. The Internet and social media were the primary information sources. This study resonates with a call to action, a call to address knowledge gaps, shape positive attitudes, and dismantle barriers surrounding emergency contraception among Syrian men. Through targeted educational interventions, heightened awareness efforts, and improved training for healthcare providers, we have the potential not only to elevate individual awareness and decision-making but also to usher in transformative improvements in reproductive health outcomes for Syrian men and their partners.
